# Implementation of a Parallel Protein Structure Alignment Service on Cloud

**DOI:** 10.1155/2013/439681

**Published:** 2013-03-25

**Authors:** Che-Lun Hung, Yaw-Ling Lin

**Affiliations:** ^1^Department of Computer Science and Communication Engineering, Providence University, 200, Sec. 7, Taiwan Boulevard, Shalu Dist., Taichung 43301, Taiwan; ^2^Department of Computer Science and Information Engineering, Providence University, 200, Sec. 7, Taiwan Boulevard, Shalu Dist., Taichung 43301, Taiwan

## Abstract

Protein structure alignment has become an important strategy by which to identify evolutionary relationships between protein sequences. Several alignment tools are currently available for online comparison of protein structures. In this paper, we propose a parallel protein structure alignment service based on the Hadoop distribution framework. This service includes a protein structure alignment algorithm, a refinement algorithm, and a MapReduce programming model. The refinement algorithm refines the result of alignment. To process vast numbers of protein structures in parallel, the alignment and refinement algorithms are implemented using MapReduce. We analyzed and compared the structure alignments produced by different methods using a dataset randomly selected from the PDB database. The experimental results verify that the proposed algorithm refines the resulting alignments more accurately than existing algorithms. Meanwhile, the computational performance of the proposed service is proportional to the number of processors used in our cloud platform.

## 1. Introduction

Protein structure alignment is a useful strategy for structural biology. Most of the alignment methods rely on structure comparison to identify structural, evolutionary, and functional relationships between proteins [1]. In general, these methods align proteins based on structural similarity. A structural alignment can identify the evolutionary equivalent residues when the aligned proteins share a common ancestor. Unlike sequence alignment tools, which focus on equivalent residues, structural alignment methods focus on conserved protein structure. Therefore, structural alignments of remote homologous proteins are more reliable than sequence alignments. Structural alignment identifies functional mechanisms by comparing functionally related proteins and can also annotate the function of proteins whose structures have been detected.

Several protein structural alignment methods [2–8] compare protein structures by structural similarity based on secondary structure elements, as well as intra- and intermolecular atomic distances. The basic idea of structure alignment is to identify the secondary structural elements, cluster these elements into groups, and score the best substructure alignment. The Vector Alignment Search Tool (VAST) [2] compares protein structures according to the continuous distribution of domains in the fold space. VAST has been used to compare all known Protein Data Bank (PDB) domains to each other. The alignment results are presented in NCBI's Molecular Modeling Database [9].

DALI [3] aligns proteins using several 2D distance matrices that represent all intramolecular distances between the C*α* atoms. It splits the protein sequences into hexapeptide fragments and calculates 2D distance matrices by measuring the contact patterns between consecutive fragments. The similarity search is conducted through a series of overlapping submatrices. The most similar sub-matrices are reassembled into the final alignment. DALI was used to create the FSSP database from 3D structure comparisons of protein structures from PDB. DALI is also responsible for automatic maintenance and update of the FSSP database. The combinatorial extension (CE) method [5] operates similarly to DALI, in that each protein sequence is fragmented. These fragments are then reassembled into a complete alignment. A final alignment is calculated as the optimal path through the similarity matrix and is extended with the next highest-scoring aligned fragment pairs. GANGSTA+ [6] aligns nonsequential structural protein sequences and performs similarity searches of databases. This algorithm adopts a combinatorial approach to evaluate secondary structural similarities between two protein structures based on contact maps. SSAP [7] uses a dynamic programming approach based on atom-to-atom vectors in the structure space. Different to other dynamic programming methods, SSAP adopts a double dynamic programming strategy. SPalign [8] is a pairwise protein structure alignment method that compares protein sequences using a size-independent scoring function called SPscore, which can fix the cutoff distance at 4 Å. Another parameter, the normalization prefactor, omits the size dependence. Improvements to structure alignment methods have been actively researched, and new or modified methods have become widely distributed web services. Increasing numbers of protein structure alignment tools are being deployed online, enabling users to submit their data and obtain the final alignments on websites [2, 3, 6, 8]. 

The recently developed service deployment model called cloud computing can deliver computing resources, either hardware or software, via the internet. The cloud computing platform relies on virtualization technology to concentrate all physical resources into a large resource pool. Virtualization allows users to access desired resources from the cloud computing environment. Hadoop [10] is a software framework designed to support data-intensive distributed applications. It can process petabytes of data through thousands of nodes. Hadoop supports a parallel programming model, called MapReduce [11], that enables parallelization of large datasets. MapReduce possesses several important characteristics; namely, high availability, scalability, and fault tolerance. In traditional parallel programming models such as MPI, OpenMP, and Pthread, a computation job is interrupted when a node in the cluster system fails. MapReduce can recover the failed computation job by reassigning the job to healthy nodes. Recently, Hadoop has been applied in several bioinformatics domains [12–14]. CloudBurst [12] is a parallel algorithm that maps next-generation sequence data to reference genomes. This algorithm has been adopted in researches such as SNP discovery, genotyping, and personal genomics. Sudha Sadasivam and Baktavatchalam [13] proposed a Hadoop-based multiple sequence alignment method to solve large-scale alignment problems. Another Hadoop-based system, Crossbow [14], is a scalable, portable, and automatic cloud computing tool that detects SNPs among short read data. 

In this paper, we propose a cloud service for protein structure alignment. The service is implemented in the Hadoop framework on a virtualization cloud platform. Structural alignment methods based on atom-pairing schemes, such as VAST, CE, and DALI, require a reliable isometric transformation by which to produce the best atom-pairing alignment between two proteins. Therefore, we introduce a refinement algorithm that uses isometric transformations to compare two protein structures. The algorithm refines the output of existing structural alignment methods such as VAST. In our cloud service, the protein structure alignment and refinement algorithms are executed under the Map/Reduce framework. The Map/Reduce framework is performed in the virtualization cloud environment. By comparing the proposed algorithm with existing protein structural alignment tools, we demonstrate the superior accuracy of our approach. In addition, the computational performance of the proposed service can be enhanced proportionally to the number of Hadoop Map operations. The cloud service is available at http://bioinfo.cs.pu.edu.tw/bioinfo/.

## 2. Materials and Methods

### 2.1. Protein Structure Alignment and Refinement

Protein structure alignment detects homologous polymer structures based on shape and three-dimensional conformation. Protein structural alignment tools detect the evolutionary relationships between proteins by comparing proteins with low sequence similarity. In general, the outputs of a structural alignment tool are a superposition of the atomic coordinate sets and the minimum root mean square deviation (RMSD) between the structures. The RMSD of two aligned structures indicates their divergence from each other. Therefore, RMSD measures the accuracy of the structural alignments. The smaller the RMSD value the more accurate the structural alignment. The RMSD is defined below. 

Let **T** = (*t*_1_,…, *t*_*n*_) and **L** = (*l*_1_,…, *l*_*n*_) be two sequences of points. The *i*th (*X*, *Y*, *Z*) coordinate value of a point in *x* is denoted by *x*_*i*_, and |*x*| denotes the length of *x*. Let *d* be the RMSD function which produces the RMSD value, then

(1) d(T,  L,  R,  δ)=1n∑k=1n|Rtk+δ−lk|2,

where **R** is a rotation matrix and **δ** is a translation vector. The minimum RMSD value **d**(**T**, **L**) between **T** and **L** is defined as **d**(**T**, **L**) = min⁡{**d**(**T**,  **L**,  **R**,  **δ**)}.

The proposed cloud computing service for protein structure alignment comprises two main stages: structural alignment and alignment refinement. The refinement strategy adopts two approaches, minibipartite and parametric adjustment. The proposed protein structure alignment is operated as follows.

*Stage* 1: *Protein Structure Alignment*. The first task of the proposed cloud server is to structurally align the proteins. Our platform uses two widely used protein structure alignment algorithms, DALI [2] and VAST [3]. The produced alignment is then input to the refinement strategy.

**Figure 1:**
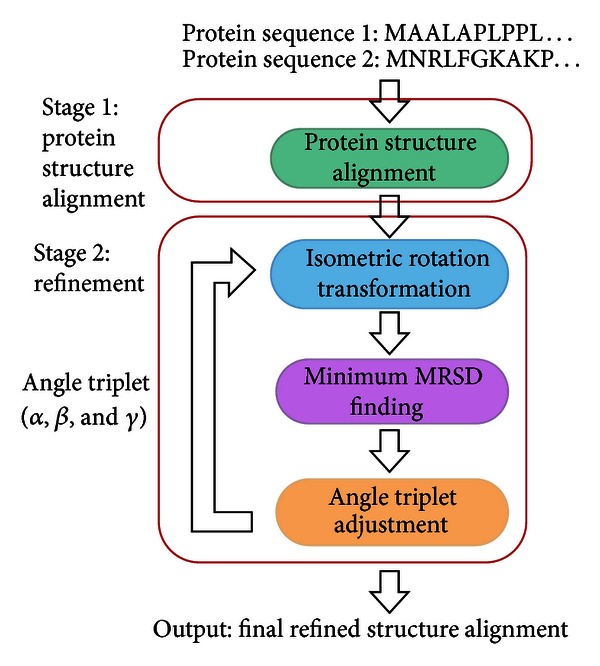
Procedure of the refinement stage.

*Stage* 2: *Refinement. *The proposed cloud service not only provides structural alignment but also develops a refinement algorithm to reduce the RMSD of the original alignment. This stage consists of three steps: isometric rotation transformation, minimum bipartite matching, and angle triplet adjustment, as described below. The refinement procedure is illustrated in Figure 1.

(i) *Isometric Rotation Transformation. *The parameter input to the RMSD scoring function is the rotation matrix **R**. To achieve a small RMSD score, this rotation matrix must be provided in a protein structure alignment. Euler's rotation theorem [15] states that any rotation about the origin can be expressed as three angular parameters. A rotation matrix is defined in terms of two axes (*x*, *z*) and three Euler angles (*α*, *β*, and  *γ*). Firstly, angle *α* rotates around the *z*-axis; next, angle **β** rotates around the *x*-axis, followed by a third rotation through angle *γ* around the *z*-axis.

Given a unit vector **n** = (0,0,1)^*T*^, and the rotation matrix, **R**, **n** is rotated to another unit vector **p** = (*x*,*y*,*z*)^*T*^, that is, **p** = **R** **n**. Two angles, *α* and *β*, determine the *z*-coordinate of **p** and the *x*- and *y*-coordinates of **p**, respectively. The number of rotations is unlimited. A rotation **R** can be made by rotating all other points around the vector **p** by the angle *γ*. In general, a rotation transformation is parameterized by an angle triplet (*α*, *β*, and *γ*). Thus, a vector **p** = (*x*,*y*,*z*)^*T*^ on the surface of the unit sphere is a probe. Each probe is shifted from vector (0,0,1)^*T*^ to other points within the sphere. The position of **p** is decided by two angles (*α*, *β*), and its rotation is decided by the angle *γ*. 

The rotation matrix is characterized by adjusting the three distributed angles (*α*, *β*, and *γ*). Similar to Euler's rotation transformation, the rotation through the angle triplet (*α*, *β*, *γ*) is achieved as follows. 

*First Rotation.* Given a unit vector **p** = (*x*,*y*,*z*)^*T*^, **p** is transformed into **p**′ by rotating the *z*-axis through angle *α*. **p**′ = (*x*_*α*_,*y*_*α*_,*z*_*α*_)^*T*^. More precisely

(2) p′=[C1S10−S1C10001]·p,

where *C*_1_ and *S*_1_ denote cos⁡*απ* and sin*απ*, respectively.

*Second Rotation*. The vector **p**′ is transformed into the probe **p**′′ by rotating an angle **β** around the *x*-axis with **p**′′ = (*x*_*β*_,*y*_*β*_,*z*_*β*_)^*T*^; more precisely

(3) $$\begin{array}{c} p^{\prime \prime }=\left[\begin{array}{ccc} 1 & 0 & 0\\ 0 & C_{2} & -S_{2}\\ 0 & S_{2} & C_{2} \end{array}\right]\cdot P^{\prime }, \end{array}$$

where *C*_2_ and *S*_2_ denote cos⁡*βπ* and sin*βπ*, respectively.

*Third Rotation.* The rotation matrix **R** is obtained as a rotation around **p**′′ by angle **γ** [16]. That is,

(4) R=[C3+x2hxyh−zS3xzh+yS3xyh+zS3C3+y2hyzh−xS3xzh−yS3yzh+xS  3  C3+z2h],

where *C*_3_ and *S*_3_ denote cos*γπ* and sin*γπ*, respectively, and *h* = 1 − cos⁡*γπ*.

The rotation matrix **R**, which determines the RMSD value, is calculated after three self-rotations in the above example. Since the number of rotations is unlimited, many RMSD values can be computed from **R**s calculated by various sets of unit vectors. 

(ii) *Minimum RMSD Finding.* Since smaller RMSD value implies higher structural alignment accuracy, the proposed refinement algorithm seeks an alignment that minimizes the RMSD. The minimum bipartite matching algorithm identifies the two sets of unit vectors with the smallest RMSD value. We adopt the Munkres [17, 18] algorithm in this step. Let **P**′ and **Q**′ be translated from **P** and **Q**, respectively. The mass centers of **P**′ and **Q**′ remain at their respective original locations **P** and **Q**. Giving a weighed graph *G* = (*V*, *E*), *V* is labeled with points of **P**′ and **Q**′, and each (*p*, *q*) in *E* is weighted by the squared Euclidean distance. The RMSD of the final alignment is reduced by pair matching.

(iii) *Angle Triplet Adjustment. *The RMSD values and unit vectors are related through the isometric rotation transformation formula. Although minimum bipartite matching identified the smallest RMSD values from various rotations, the RMSD is reduced further by adjusting unit vectors with angle triplets. In this step, angle triplets are adjusted by trigonometric series to form different unit vectors.

Trigonometric series can approximate the angle triplets with smaller RMSD values. The angles *α*, *β*, and *γ* are sequentially adjusted, and the evaluation function *f*(*θ*) corresponds to the RMSD values altered by the adjustments. The *f*(*θ*) is defined as follows:

(5) $$\begin{array}{c} f\left(\theta \right)=C_{1}+\left(\sum_{i=2}^{k}C_{i}\cos i\pi \theta +C_{i+1}\;\sin i\pi \theta \right), \end{array}$$

where *k* is the number of local maximum vectors and *θ* = (*α*, *β*, *γ*). The adjusted angles evaluated by *f*(*θ*) constitute the new parameters in the isometric rotation transformation. The refinement step is performed iteratively until degree *k* is reached. 

Most existing alignment tools are computationally time consuming and are best implemented under powerful parallel processing. Moreover, the user expects that the computational alignment process never fails. Therefore, fault tolerance and high availability are important issues in current computational services.

### 2.2. Cloud Computing Platform

The proposed cloud computing platform combines two technologies: the Hadoop framework and virtualization. The protein structure alignment and the proposed refinement algorithm are implemented in Hadoop and are deployed on a virtualized computing environment. 

**Figure 2:**
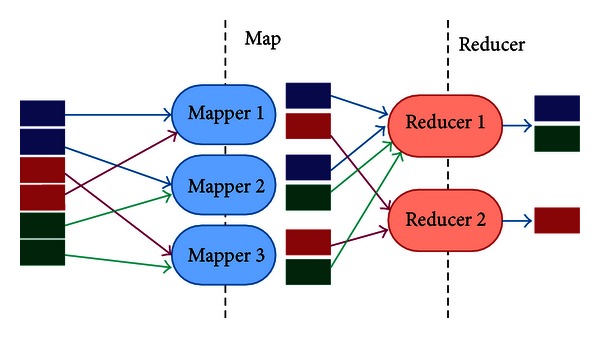
Implementation of Hadoop Map/Reduce model.

Hadoop is a distribution computation framework that coordinates computing nodes for parallelized data distribution. It adopts the two-layer Map/Reduce parallel programming model. Many cloud computing vendors, such as Yahoo, Amazon EC2, IBM, and Google, have supported the Map/Reduce model. An application implemented by this model comprises Map and Reduce stages, as shown in Figure 2. The input data is first split into smaller chunks corresponding to the number of Mappers. Each Mapper processes an allocated data chunk. Map stage data are output as <key, value> pairs. The <key, value> pairs are classified by key and are assigned to a corresponding Reducer. In the Reduce stage, the Reducer sums all values belonging to the same key among the assigned <key, value> pairs. The Reduce stage outputs <key, value> pairs, where each key is unique. 

A Hadoop cluster includes a single master and multiple slave nodes. The master node consists of a job tracker, task tracker, name node, and data node. A slave node, or computing node, comprises a data node and task tracker. The job tracker and the task-tracker execute the Map/Reduce stages. Data are stored in the name node and the data node. The job tracker distributes Map/Reduce tasks to specific nodes in the cluster, ideally to those nodes already containing the data, or at least within the same rack. A task tracker is a node in the cluster that accepts Map, Reduce, and Shuffle operations from a job tracker.

Hadoop Distributed File System (HDFS) is the primary file system used by the Hadoop framework. Each input file is split into data blocks that are distributed to data nodes. Hadoop evades faults by creating multiple replicas of data blocks and distributing them to data nodes throughout a cluster, thereby enabling reliable, extremely rapid computations. The name node manages a directory namespace and a node metadata for the HDFS. A Hadoop cluster operates on a single name node.

Virtualization in the cloud computing environment ensures efficient use of the physical resources. The physical resources, including computing power, storage and network, are regarded as utilities that users can pay for as required. The usual goal of virtualization is to improve scalability and overall hardware-resource utilization. Virtualization enables the simultaneous running of operating systems in a single physical computer. While a physical computer constitutes a complete and actual machine, a virtual machine (VM) is a completely isolated machine running a guest operating system within the physical computer. All nodes within a Hadoop cluster of the proposed cloud service, such as job tracker, task tracker, name node, and data nodes, operate in virtual machines. 

**Figure 3:**
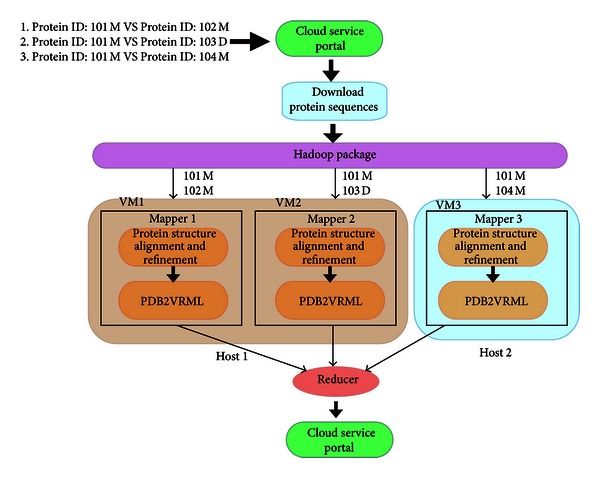
The architecture of the proposed cloud computing service.

The architecture of the proposed cloud computing service is illustrated in Figure 3. All mappers and Reducers work in virtual machines. The service accepts PDB ID as input data. The wwPDB (Protein Data Bank) [19] is a widely accessed database that archives experimentally determined structures of proteins, nucleic acids, and complex assemblies. The PDB ID identifies a specific protein structure. The submitted PDB ID pair is stored in a job queue file. Assuming that *N* task trackers must distribute *P* PDB ID pairs in the job queue file, the *i*th line in the queue file will as assigned as the *i*th map task and sent to Hadoop by streaming operation. Each task-tracker node receives a map task which aligns the protein structure and executes the refinement algorithm. The refined alignment is converted to a 3D protein structure image using the PDB2VRML tool [20]. When a task-tracker node has completed a map task, it passes the score to a Reducer and executes a new map task. Computation continues until all map tasks are complete. Generally, each task-tracker node is assigned *P*/*N* map tasks. In the proposed cloud computing service, the Reduce task that collects the RMSD value of each PBD ID pair is performed solely by the Reducer. Finally, the Reducer stores the RMSD values in a file by HDFS.

## 3. The Cloud Computing Platform

The proposed cloud computing service for protein structure alignment can be regarded as BaaS (Bioinformatics as a Service). The proposed service, accessible through the internet, enables molecular biologists to efficiently execute 3D protein structure alignment. Supplied with two user-input PDB IDs, the service searches protein structure data archived in the wwPDB and compares the protein structures using Hadoop. 

**Figure 4:**
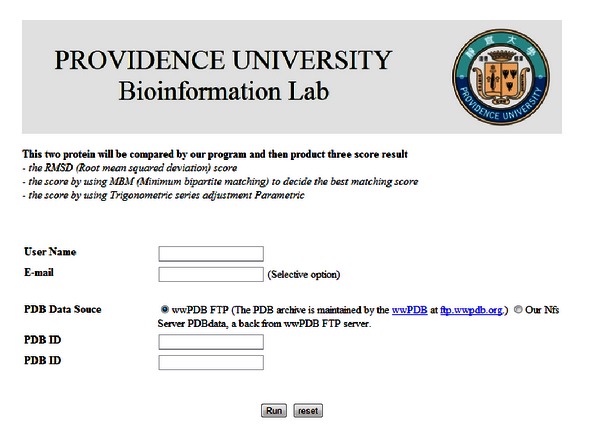
Cloud service portal.

**Figure 5:**
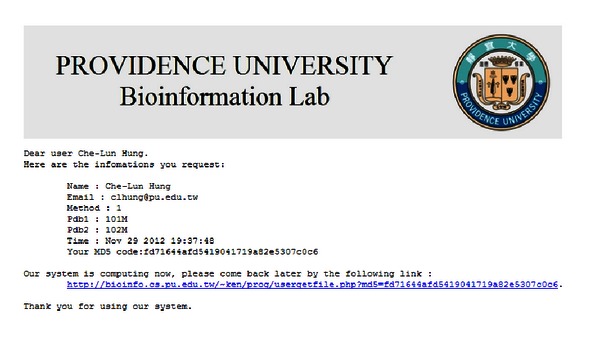
The webpage indicates a submitted request of protein structure alignment.

**Figure 6:**
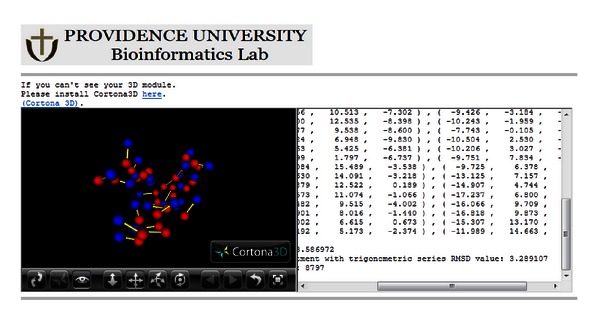
Protein structure alignment and 3D structural image produced by the cloud service.

The proposed service provides users with a hyperlink for accessing the alignment result before the computation is complete. In this way, the user can repeatedly view and download the result. This hyperlink is accessible either from the website or by email. The portal of the proposed service is illustrated in Figure 4.  Figure 5 shows the submitted job information, including the hyperlink enabling result download.  Figure 6 shows the output of the proposed service, including a 3D structural image of the protein [21] and RMSD values. 

## 4. Experiment

The experimental computing environment comprises an NFS server and four IBM blade servers. Each server is equipped with two Quad-Core Intel Xeon 2.26 GHz CPUs, 24 GB RAM, and 296 GB hard drive. Under the current system environment, we create 8 virtual machines by Kernel-based Virtual Machine (KVM); each virtual machine is set to one single-core CPU, 2 GB RAM, and 30 GB hard drive and runs Hadoop version 0.2. Each virtual machine is responsible for a map operation and a Reduce operation. Therefore, up to 8 Map/Reduce operations are possible.

Protein structure data sources used in the experiments were downloaded from the World Wide Protein Data Bank (http://www.wwpdb.org/). The PDB ID consists of 4 letters. The protein data bank contains 80,402 protein structures, from which 1000 protein pairs were selected as test data by uniform-random sampling.

First, we evaluated the improvement of RMSD values by the proposed refinement algorithm. Structure alignment was undertaken by two widely used algorithms, DALI and VAST. These alignments were input to the proposed refinement algorithm. The comparison between the original RMSD values produced by DALI and VAST and the refined values of our proposed algorithm is summarized in Table 1. The RMSD values produced by DALI and VAST were improved using isometric rotation transformation and bipartite matching. The improved alignments can also be improved in advance by angle triplet adjustment, as seen in Table 1. Our approach improved the RMSD values of DALI and VAST by approximately 7% and 6%, respectively. Clearly, the proposed refinement algorithm can significantly improve the RMSD values produced by standard protein structure alignment methods. 

**Figure 7:**
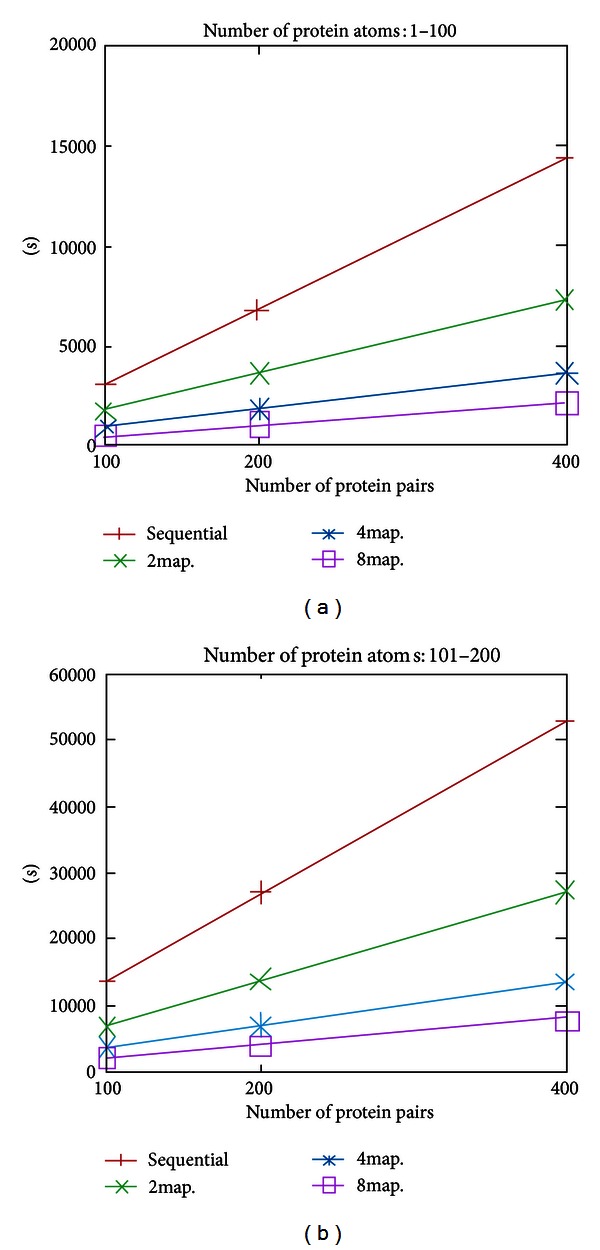
RMSD values produced by various numbers of Mappers.

To assess the performance of the proposed cloud service based on the Hadoop framework, the execution time of the service was compared for varying structural data size and number of Map/Reduce operations.  Figure 7 illustrates the performance of the proposed service under the MapReduce framework. The execution time is effectively reduced when more map operations are deployed. Compared to the sequential algorithm (implemented in the proposed service with a single mapper), introduction of two, four, and eight mappers improved the execution time by approximate factors of two, four and eight. The computation efficiency is improved by an amount proportional to the number of mappers, although the execution time increases as the number of protein pairs and protein atoms increases (see Figure 7). We infer that the Hadoop framework significantly reduces the computational cost. 

## 5. Conclusion

Identifying the evolutionary relationship between proteins has become reliant on protein structure alignment. Several online alignment tools are currently available for comparing protein structures. These methods are widely used in bioinformatics, but their implementation on a single computer limits their computing power and data availability. To remedy this situation, we propose a novel biocloud service for protein structure comparison based on virtualization technology and the Hadoop framework. We also propose an algorithm for refining the alignment produced by standard protein structural alignment tools such as DALI and VAST. The algorithms are integrated with the Hadoop parallel computing platform. Our service provides molecular biologists with a high performance, fault tolerant, and high-availability protein structure analysis platform. The proposed cloud service was experimentally verified as suitable for investigating protein structure functions.

In future work, we will investigate an automatic deployment model that dispenses bioinformatics tools as cloud computing services. The Hadoop framework and virtualization technology ensures high performance in a robust computing environment. Due to the scalability of our platform, it can adequately process increasingly vast quantities of bioinformatics data. 

## Figures and Tables

**Table 1:** RMSD computed by our proposed algorithm, DALI, and VAST.

Protein structure alignment methods	Original RMSD value	The average RMSD value refined by bipartite matching	The average RMSD value after the proposed refinement algorithm
DALI	1.5767	1.5166	1.4713
VAST	1.5114	1.4664	1.4228

## References

[1] Gerstein M, Jansen R, Johnson T, Tsai J, Krebs W (1999). Motions in a database framework: from structure to sequence. *Rigidity Theory and Applications*.

[2] Gibrat JF, Madej T, Bryant SH (1996). Surprising similarities in structure comparison. *Current Opinion in Structural Biology*.

[3] Holm L, Sander C (1998). Touring protein fold space with Dali/FSSP. *Nucleic Acids Research*.

[4] Orengo CA, Michie AD, Jones S, Jones DT, Swindells MB, Thornton JM (1997). CATH—a hierarchic classification of protein domain structures. *Structure*.

[5] Shindyalov IN, Bourne PE (1998). Protein structure alignment by incremental combinatorial extension (CE) of the optimal path. *Protein Engineering*.

[6] Guerler A, Knapp EW (2008). Novel protein folds and their nonsequential structural analogs. *Protein Science*.

[7] Taylor WR, Flores TP, Orengo CA (1994). Multiple protein structure alignment. *Protein Science*.

[8] Yang Y, Zhan J, Zhao H, Zhou Y (2012). A new size-independent score for pairwise protein structure alignment and its application to structure classification and nucleic-acid binding prediction. *Proteins*.

[9] NCBI's Molecular Modelling Database http://www.ncbi.nlm.nih.gov/Structure/MMDB/mmdb.shtml.

[10] Hadoop—Apache Software Foundation project. http://hadoop.apache.org/.

[11] Taylor RC (2010). An overview of the Hadoop/MapReduce/HBase framework and its current applications in bioinformatics. *BMC Bioinformatics*.

[12] Schatz MC (2009). CloudBurst: Highly sensitive read mapping with MapReduce. *Bioinformatics*.

[13] Sudha Sadasivam G, Baktavatchalam G (2010). A novel approach to multiple sequence alignment using hadoop data grids. *International Journal of Bioinformatics Research and Applications*.

[14] Langmead B, Schatz MC, Lin J, Pop M, Salzberg SL (2009). Searching for SNPs with cloud computing. *Genome Biology*.

[15] Euler L (1775). Formulae generales pro translatione quacunque corporum rigidorum. *Novi Commentarii academiae scientiarum Petropolitanae*.

[16] Grap A (1918). *A Treatise on Gyrostatics and Rotational Motion*.

[17] Munkres J (1957). Algorithms for the assignment and transportation problems. *Journal of the Society for Industrial and Applied Mathematics*.

[18] Bourgeois F, Lassalle JC (1971). Extension of the Munkres algorithm for the assignment problems to rectangular matrices. *Communications of the ACM*.

[19] wwPDB http://www.wwpdb.org/.

[20] PDB2VRML Library http://www.oocities.org/gnubioq/pdb2vrml/.

[21] Cortona 3D Viewer http://www.cortona3d.com/Products/Viewer/Cortona-3D-Viewer.aspx.

